# Endometrial Stromal Sarcoma Recurrence in the Caecum

**DOI:** 10.1155/2018/9139281

**Published:** 2018-08-05

**Authors:** Nisar A. Chowdri, Asif Mehraj, Fazl Q. Parray, Mudassir A. Khan, Masood A. Laharwal, Rauf A. Wani

**Affiliations:** ^1^Department of Colorectal Surgery, SKIMS, Srinagar, J&K, India; ^2^Department of Neurosurgery, SKIMS, Srinagar, J&K, India

## Abstract

Endometrial stromal sarcomas of uterus are quite rare. Most of the recurrences in these tumors are seen in the pelvis. However, extrapelvic recurrences and metastases to other parts are quite unusual. Here, we are reporting a rare case of caecal recurrence of endometrial stromal sarcoma. *Case Report*. A 52-year-old female presented to us with pain and lump in the right lower abdomen. The patient was earlier subjected to total abdominal hysterectomy with bilateral salpingo-oophorectomy (TAHBSO) for low-grade endometrial stromal sarcoma. Postoperatively patient received radiotherapy but no hormone therapy. After 10 years of follow up patient presented with a polypoidal lesion in the caecum. Patient was evaluated fully and subjected to resection of this polypoidal lesion, which proved out to be high-grade endometrial stromal sarcoma. *Conclusion*. Recurrence of endometrial stromal sarcoma in the caecum is very rare. However, this entity needs to be kept in mind for differential diagnosis of a caecal mass. Recurrence in such cases may present quite late.

## 1. Introduction

Endometrial stromal sarcomas (ESS) are a rare variant of malignant mesenchymal tumors. This disease entity belongs to the rarest uterine malignancies (prevalence category < 1–9/1,000,000) [1]. These usually develop in the uterine corpus and may occasionally arise at various extrauterine sites. World Health Organization has classified ESS into three categories: low-grade endometrial stromal sarcoma (LG-ESS), high-grade endometrial stromal sarcoma (HG-ESS), and undifferentiated uterine sarcoma (UUS) [2]. Recurrences at distant sites are known and reported in the literature [3]. We present a case of atypical recurrence from such a tumor not reported in the literature till date.

## 2. Case Report

**Figure 1:**
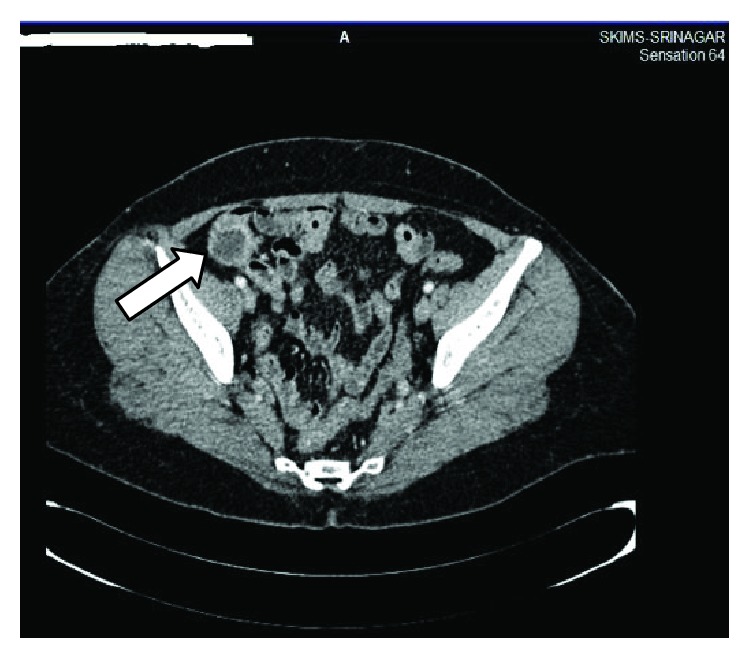
Well-defined lesion, anteromedial to the right external iliac vessels.

A 52-year-old female was subjected to total abdominal hysterectomy with bilateral salpingo-oophorectomy (TAHBSO) for LG-ESS. Patient received radiotherapy and was doing well for 10 years. After 10 years, she presented with the right lower abdominal discomfort and a lump. USG showed an ill-defined mass with heterogenous echogenicity in the right lower quadrant of the abdomen. MRI revealed a well-defined regular contour lesion, measuring 5.3 × 4.8 cm with isointense signals on T2-weighted images, anteromedial to right external iliac vessels (Figure 1). Lesion showed significant diffusion restriction on diffusion-weighted images.

**Figure 2:**
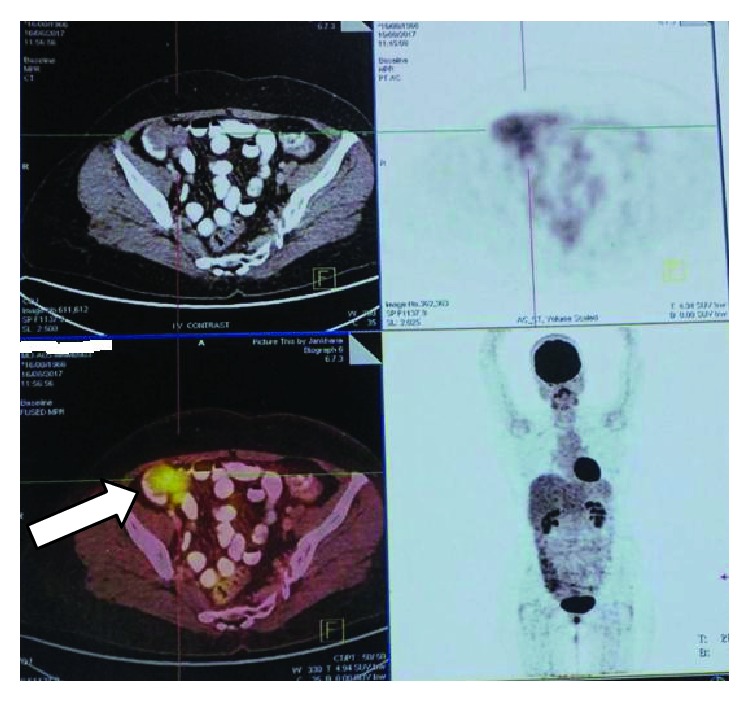
PET/CT scan showing well-defined heterogeneously enhancing lesion in the right iliac fossa along the anterior abdominal wall.

PET/CT scan (Figure 2) showed a well-defined heterogeneously enhancing lesion in the right iliac fossa abutting the adjacent bowel loops measuring approximately 4.9 × 5.1 cm. No abnormal enhancing lesion or abnormal metabolic activity was seen at the operative site.

**Figure 3:**
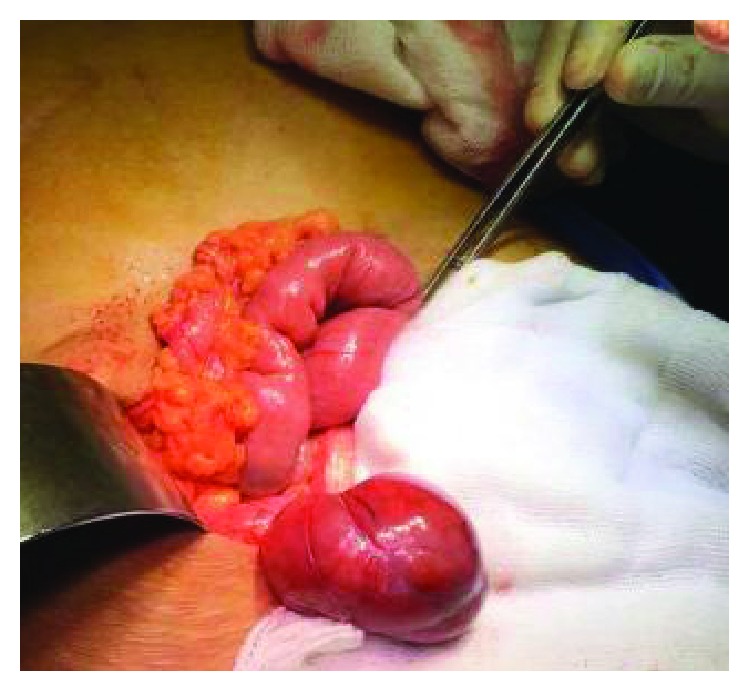
Intraoperative picture showing pedunculated mass arising from the caecum.

Tumor markers (Ca125 and CEA) and other baseline investigations were within normal limits. Operative findings revealed 5 × 4 cm solid thin pedicled, well encapsulated, and mobile mass arising from the caecum, without being adherent to surrounding bowel loops or other structures (Figure 3). Previous operative site and rest of the pelvis was free of any deposits. Wide local excision with partial caecectomy, appendectomy, and omental biopsy was done. Patient had an uneventful postoperative period and was discharged on the 3rd postoperative day.

**Figure 4:**
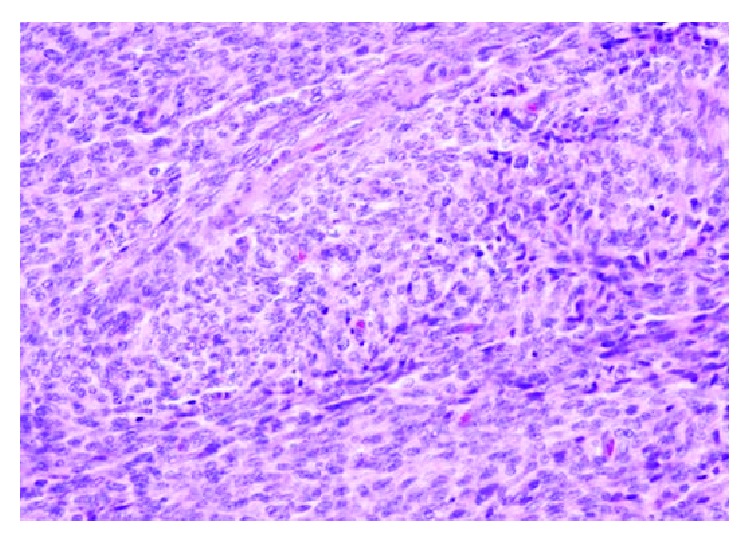
Hematoxylin and eosin stain of caecal endometrial stromal sarcoma, showing round to oval spindle-shaped nuclei with nuclear atypia.

**Figure 5:**
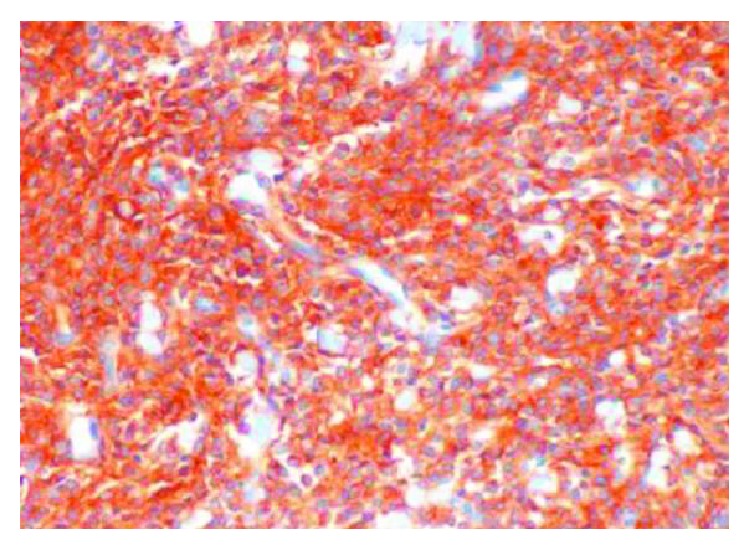
Immunohistochemistry picture showing CD10 positivity.

**Figure 6:**
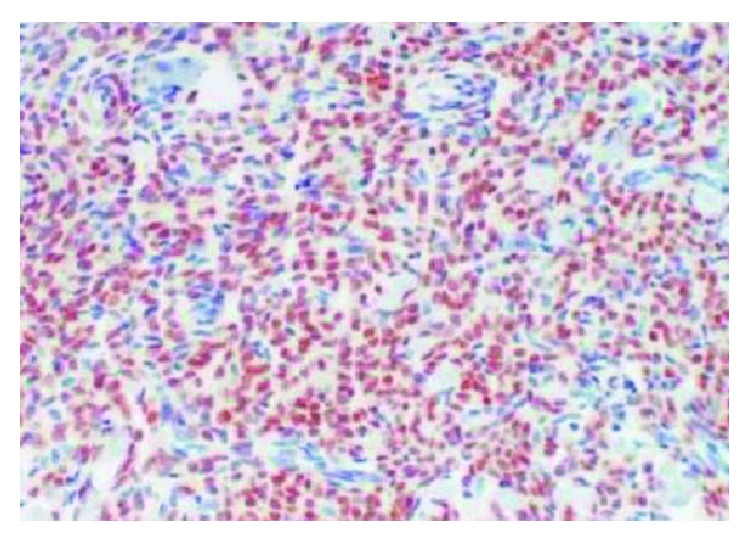
Immunohistochemistry picture showing estrogen receptor positivity.

**Figure 7:**
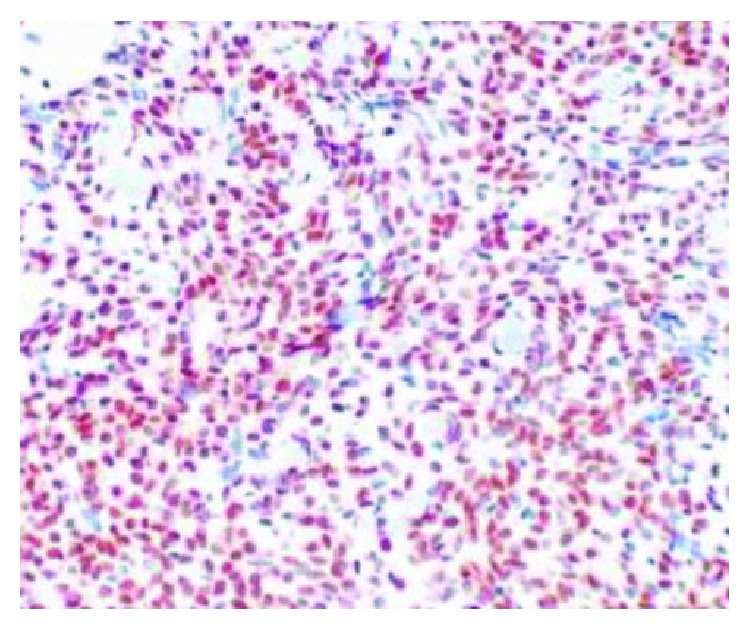
Immunohistochemistry picture showing progesterone receptor positivity.

Histopathological examination revealed features of high-grade endometrial stromal sarcoma (Figure 4). Immunohistochemistry revealed CD10 (Figure 5), estrogen receptor (ER, Figure 6), and progesterone receptor (PR, Figure 7) positivity. Patient received postoperative hormone therapy and was recurrence-free on a follow-up of more than 1 year.

## 3. Discussion

Low-grade endometrial stromal sarcomas (LG-ESS) are usually slow-growing neoplasms with an indolent clinical course, whereas high-grade ESS often show more aggressive nature. For LG-ESS, total abdominal hysterectomy is recommended as primary treatment, whereas the need for bilateral salpingo-oophorectomy, adjuvant chemotherapy, radiotherapy, or hormonal therapy is still controversial [4]. Our patient underwent a total abdominal hysterectomy with bilateral salpingo-oophorectomy (TAHBSO) for menorrhagia. LG-ESS were an incidental finding on histopathological examination with tumor limited within the serosa, meaning that the tumor was an early-stage tumor and unlikely to produce tumor seedlings.

Recurrence develops in one-third to one-half of patients with LG-ESS. It has been reported even 30 years after the initial treatment [5] and usually includes multiple lung metastases, peritoneal metastases, and/or local recurrences [3]. Recurrence may be attributed to growth stimulus to residual tumor cells by estrogen. After oophorectomy, estrogens produced by peripheral tissues or exogenous administration in the form of hormone replacement therapy may be a reason for recurrence [6]. There is currently no standard therapy for patients with recurrent disease. Various treatment modalities like hormone therapy, radiotherapy, surgical reexcision, or a combination of these have been used to treat recurrent ESS [7]. Chu et al. [5] showed that 75% of patients with stage I disease did not recur if treated with adjuvant megestrol compared to 29% of similarly staged patients who did not receive adjuvant megestrol. He concluded that in relapsing patients, progestin therapy along with surgery may be responsible for prolonged complete response. The author suggested megestrol acetate 160 mg daily for adjuvant therapy for stage I patients and maintenance of this regimen for 2 years. For advanced stage patients or those with recurrent disease, the maintenance of this dosage is suggested indefinitely. Maluf et al. reported that progestin therapy may have a positive effect on the disease, causing the inhibition of endometrial epithelial proliferation [8].

Li and Chang [9] reported a case, in which a 41-year-old patient developed recurrence of ESS in the caecum, 11 years after surgical treatment. She presented with ileocolic intussusception and was subjected to right hemicolectomy. The patient was then treated with progestin for 2 years and did not report any recurrence for 2 years. They emphasized the importance of long-term follow-up and initial surgical treatment of ESS and suggested systemic hormonal therapy in recurrent cases. Our patient also presented with recurrence of ESS in the caecum, but her clinical presentation was discomfort and a lump in the right lower abdomen. So the presentation of such a lesion can vary from mild pain to signs of bowel obstruction.

ESS may arise as a primary extrauterine endometrial sarcoma (EESS), mainly on the background of endometriosis, with predominant gonadal involvement. Very rarely does EESS arise primarily in the gastrointestinal or extragastrointestinal tract organs outside the pelvis or evolve without preceding endometriosis [10]. Our patient had no previous history of endometriosis of the pelvis or bowel and did not present as a recurrence at the previously operated site. Presentation was atypical in a way that the recurrence was late and at an unusual site with high-grade histology. It is unlikely to be a different tumor as there was no evidence of endometriosis or use of any exogenous hormones.

The main differential diagnosis for EESS when arising from bowel is some other mesenchymal tumor, especially gastrointestinal stromal tumor (GIST). Spindle cells in EESS are characterized by monotonous arrangement, compared with sheets or fascicle arrangement in GIST. Proliferation of small arterioles, resembling spiral arterioles of the endometrium, is also typical of EESS. Mucocele of appendix can also present in this manner. However, the final diagnosis, especially in cases of unusual tumor location and absence of endometriosis, depends on immunohistochemistry. Positive labelling for CD10, PR, and ER with no reactivity for CD117 and CD34 confirms the diagnosis of ESS [10–12]. Diagnosis in our patient was also confirmed by CD10, ER, and PR positivity on immunohistochemistry.

Recurrence of endometrial stromal sarcoma arising in the caecum is rare. Postoperative hormone therapy is important in such cases. Besides, this entity even though rare should be kept in mind for differential diagnosis of a caecal mass.
